# General practitioners’ willingness to participate in research: A survey in central Switzerland

**DOI:** 10.1371/journal.pone.0213358

**Published:** 2019-03-01

**Authors:** Serlha Tawo, Sileni Gasser, Armin Gemperli, Christoph Merlo, Stefan Essig

**Affiliations:** 1 Department of Internal Medicine, Lucerne Cantonal Hospital, Lucerne, Switzerland; 2 Institute of Primary and Community Care Lucerne, Lucerne, Switzerland; 3 Department of Health Sciences and Health Policy, University of Lucerne, Lucerne, Switzerland; 4 Swiss Paraplegic Research, Nottwil, Switzerland; Hofstra University, UNITED STATES

## Abstract

It is difficult to enlist the participation of medical general practitioners (GPs) in research studies. We aimed to determine the willingness of GPs in central Switzerland to participate in research, and to identify factors that facilitate or hinder research participation by GPs. To that end, we conducted a postal questionnaire survey of all 268 active GPs in the canton of Lucerne. The survey explored their interest in participating in research projects (yes/no) and factors that potentially influence their participation (5-point Likert scale from “very important” to “not at all important”). We contacted all non-responders by phone. Background information of the GPs was retrieved from the database of the cantonal association of physicians. Associations between willingness to participate in research and GP’s age, gender, type and location of practice, and the perceived relevance of facilitators were investigated via multiple logistic regression. Out of 268 GPs, 115 (43%) agreed to be contacted for future research projects. Willingness was associated with age (willing: 55% ≤ 40 y vs. 33% > 60 y) and gender (44% male vs. 38% female), and to some degree with the type of practice (50% group vs. 31% single), and location (46% urban vs. 38% rural), independently from each other. Scientists should develop methods to motivate and support GPs in single and rural practices to participate so research is representative of primary care as a whole.

## Introduction

### Background

In most parts of the world, general practitioners (GPs) are the first point of contact for patients with medical problems. Complex diseases with diverse backgrounds and unique life circumstance are seen by GPs on a daily basis [1,2]. General practice comprises a broad spectrum of medical knowledge, diagnoses and treatment plans [3]. It has been shown that primary care of good quality can have a tremendous impact on health of populations [4]. Nevertheless, a trend away from primary care to more specialized care has been noted in many western countries, including Switzerland [5,6].

To achieve primary care of good quality, the provision of care has to be effective and efficient [7]. Research in primary care with the participation of as many GPs as possible plays a crucial part to achieve this goal. Generalizability of research results is higher if a high number of unselected GPs are recruited and contribute to the results. Many countries, such as Finland and Germany, are aware of this challenge and try to strengthen research in primary care [8,9]. Between 1980 and 1990, there was a fivefold increase in publications from general practice research in Australia; since then, however, the number of publications relative to other medical disciplines has declined [10]. Impeding factors were shortcomings in training, lack of protected time and a paucity of infrastructural support [11,12]. Especially in developed, industrialized countries, another factor was that academic general practice, education, and research were inadequately financed. In comparison to other European countries, such as the UK and the Netherlands [13], research activity in primary care in Switzerland is low [14].

The purpose of this study was to identify the limitations and motivating factors related to GPs’ research participation.

## Methods

### Study design and participants

We conducted a cross-sectional study with a questionnaire survey among all GPs working in the canton of Lucerne (original and translated questionnaires can be found as S1 Text and S2 Text). The survey contained nine questions on willingness to participate in research projects (yes/no); factors that potentially influence participation; interest in predefined, specific research topics according to the planned research agenda of the institute, research designs, and research methods; and on the importance of research for the future of primary care (all 5-point Likert scales from “extremely important” to “not at all important”). We also asked study participants about their own research ideas and previous research experience (free text). The questionnaire was tested by GPs associated with the Institute of Primary and Community Care Lucerne who knew the target group well. Their feedback made sure that we achieved a consistent questionnaire with clear and understandable questions.

We disseminated the questionnaire by letter post in spring 2015. GPs who did not respond were later contacted by phone and asked to provide oral feedback on the questions on willingness to participate in research projects (yes/no) and factors that potentially influence participation (5-point Likert scales). Additionally, GPs on the phone were asked about what kind of changes were needed in their practice to enable participation in research. This information was combined with free text from the written survey (on own research ideas and previous research experience) if it contained any enabling factors. When practice assistants refused transferring the call to the requested GP, they were considered as “not willing to participate in research”. Each interview was audio recorded and transcribed.

We retrieved background characteristics from the database of the cantonal physicians’ regarding GPs (age; gender), type of practice (group or single) and location (urban or rural). The definition of urban was in accordance with cities in Switzerland per the Swiss Federal Statistical Office (Luzern, Emmen, Kriens, Horw, Ebikon, Sursee).

All respondents agreed to take part in this study. According to local and international guidelines on ethics considerations in research involving human participants, this survey among physicians on their interest in research does not raise any ethical concerns. Therefore, formal ethics approval from an ethical committee was deemed unnecessary.

### Analysis

For the analysis, we combined replies by post and phone. We compared answer categories between age groups, type of practice, location, and gender. To adjust for confounding, we performed logistic regressions with willingness to participate in research as outcome and background characteristics (age, gender, type of practice, location), and the perceived relevance of facilitators (financial compensation, time resources, interesting topic, research network, further training in research) as exposure variables. Predictors available and known from the literature to be potentially related to the outcome were included. No selection procedure was applied in accordance with recommendations [15]. Interest in predefined, specific research topics, research designs, research methods, the importance of research for the future of primary care, and free-text were analyzed descriptively.

Data management and statistical analyses were conducted in Stata/SE 13.1. We report mean scores, percentage, and standard deviation (SD)for the descriptive analysis, and odds ratios (OR) for the logistic regressions, based on the available data from the survey. The app “Automatic Call Recorder” Version 4.28 was used for recordings and transcription. The anonymized dataset can be found as S1 Dataset.

## Results

Of 281 invited GPs in the database of the physicians’ association, 13 were retired or no longer working in Lucerne. Out of 268 GPs, 139 participated in the written survey (postal response rate 52%). Participants were slightly younger, more often member of a group practice, and working in urban environments, compared to non-participants (S1 Table). The remaining 129 GPs were contacted by phone.

**Fig 1:**
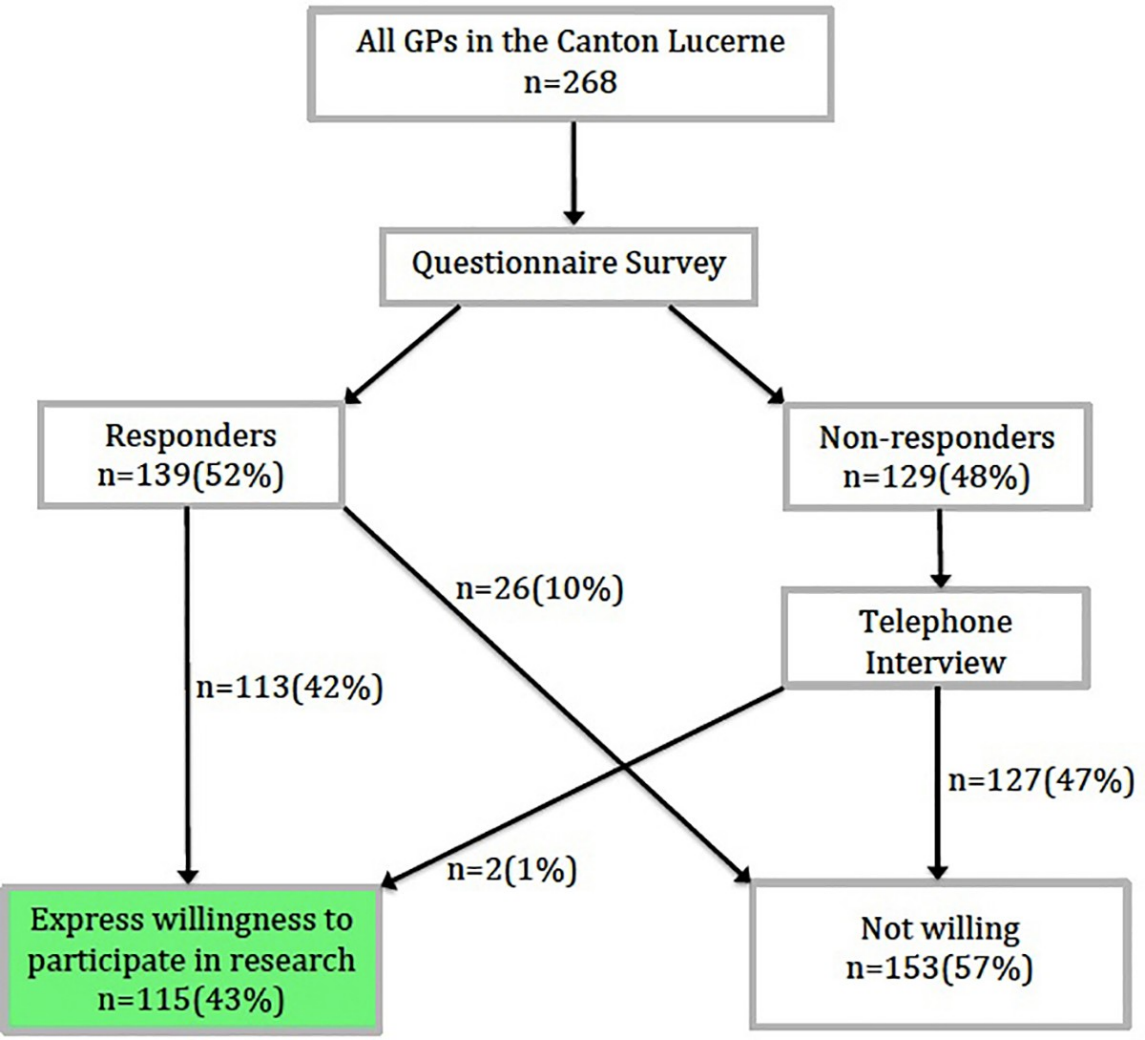
Schematic of the data collection process.

Overall, 115 (43%) of 268 GPs agreed to be contacted for research projects; 113 agreed by mail and two agreed by phone (Fig 1). Interest in research participation was stronger in younger GPs (willing: 55% ≤ 40 years) compared to older GPs (33% > 60 years), in group practices (50%) compared to single practices (31%), and in urban areas (46%) compared to rural areas (38%). Male (44%) and female (38%) GPs also differed in their willingness to participate in research, however, to a lesser degree (Table 1). When stratifying the analysis of types of practice according to location, the above-mentioned higher chance of participation in group compared to single practices (ratio 1.64) is less prominent in rural (ratio 1.31) compared to urban practices (ratio 1.90). When stratifying the analysis of location according to types of practice, the above-mentioned higher chance of participation in urban compared to rural areas (ratio 1.21) is not found in single (ratio 0.92) but group practices (ratio 1.33).

**Table 1:** Characteristics of the GPs and practices in the study population.

		Age (years)					Gender		Type of practice		Location of practice	
	n (%)	Mean	33–40	41–50	51–60	61+	Male	Female	Group	Single	Urban	Rural
**Overall**												
All	268(100%)	52.7	31(100%)	59(100%)	118(100%)	55(100%)	188(100%)	80(100%)	157(100%)	111(100%)	156(100%)	112(100%)
Willing	115(43%)	51.4	17(55%)	26(44%)	50(42%)	18(33%)	83(44%)	30(38%)	79(50%)	34(31%)	71(46%)	42(38%)
**Urban location**												
All	156(100%)	51.6	17(100%)	42(100%)	74(100%)	19(100%)	94(100%)	62(100%)	95(100%)	61(100%)		
Willing	71(46%)	50.6	10(59%)	19(45%)	33(45%)	7(37%)	45(48%)	26(42%)	53(56%)	18(30%)		
**Rural location**												
All	112(100%)	54.3	14(100%)	17(100%)	44(100%)	36(100%)	94(100%)	18(100%)	62(100%)	50(100%)		
Willing	42(38%)	52.6	14(100%)	7(41%)	17(39%)	11(31%)	38(40%)	4(22%)	26(42%)	16(32%)		
**Group practices**												
All	157(100%)	50.4	28(100%)	40(100%)	59(100%)	25(100%)	99(100%)	58(100%)			95(100%)	62(100%)
Willing	79(50%)	49.6	15(54%)	21(53%)	32(54%)	9(36%)	54(55%)	25(43%)			53(56%)	26(42%)
**Single practices**												
All	111(100%)	55.9	3(100%)	19(100%)	59(100%)	30(100%)	89(100%)	22(100%)			61(100%)	50(100%)
Willing	34(31%)	55.4	2(67%)	5(26%)	18(31%)	9(30%)	29(33%)	5(23%)			18(30%)	16(32%)

The perceived relevance of time expenses (67% “extremely important”) and selection of research topics (58%) were high; access to a research network (31%), continuous training on research questions (12%), and financial compensation (6%) were rated less important (S2 Table).

The associations of young age, urban location, and group practices with the willingness to participate in research were not sensitive to covariate adjustment. The association of male gender increased from 1.27 (95% confidence interval 0.75–2.18) to 2.04 (1.10–3.78) under adjustment. When adjusting for background characteristics and the perceived relevance of facilitators, there was a trend for those considering the facilitators very relevant to be more willing to participate in research; however, the estimates were unprecise and associations statistically insignificant (Table 2).

**Table 2:** Measures of association between exposures and willingness to participate in research.

Exposure	OR crude (95% CI)	OR adjusted for other background characteristics (95% CI)	P-value	OR adjusted for other background characteristics and perceived relevance of facilitators (95% CI)	P-value
**Age (years)**					
33–40	1	1		1	
41–50	0.65 (0.27–1.56)	0.59 (0.23–1.49)	0.268	65.7 (1.52–2845.0)	0.030
51–60	0.61 (0.27–1.34)	0.63 (0.27–1.52)	0.310	8.22 (1.00–68.04)	0.051
61+	0.40 (0.16–0.99)	0.41 (0.15–1.12)	0.082	6.17 (0.51–74.10)	0.152
**Gender**					
Female	1	1		1	
Male	1.27 (0.75–2.18)	2.04 (1.10–3.78)	0.023	8.60 (1.25–59.19)	0.029
**Location**					
Rural	1	1		1	
Urban	1.37 (0.84–2.25)	1.46 (0.85–2.51)	0.175	1.34 (0.25–7.10)	0.728
**Practice type**					
Single	1	1		1	
Group	2.41 (1.45–4.02)	2.34 (1.36–4.05)	0.002	5.47 (0.93–32.21)	0.060
**Perceived relevance of … Financial compensation**					
Very	1			1	
Quite	0.97 (0.09–9.91)			0.84 (0.32–22.39)	0.917
Undecided	0.77 (0.08–7.00)			1.34 (0.07–26.87)	0.847
Little	0.19 (0.02–1.80)			0.20 (0.01–5.75)	0.347
Least	0.11 (0.01–1.12)			0.04 (0.001–1.58)	0.087
**Time resources**					
Very	1			1	
Quite	2.92 (0.81–10.52)			58.15 (2.58–1311.3)	0.011
Undecided	0.44 (0.11–1.70)			0.09 (0.01–1.39)	0.084
Little	empty			empty	
Least	empty			empty	
**Interesting topic**					
Very	1			1	
Quite	0.66 (0.26–1.68)			0.82 (0.15–4.67)	0.826
Undecided	0.06 (0.04–1.78)			5.11 (0.21–125.9)	0.318
Little	0.60 (0.01–0.62)			empty	
Least	empty			empty	
**Research network**					
Very	1			1	
Quite	0.50 (0.14–1.77)			0.62 (0.06–5.76)	0.672
Undecided	0.33 (0.09–1.26)			0.06 (0.002–1.31)	0.074
Little	0.13 (0.02–0.70)			0.76 (0.14–42.2)	0.894
Least	0.04 (0.01–0.42)			3.73 (0.02–827.3)	0.632
**Further training in research**					
Very	1			1	
Quite	0.27 (0.03–2.32)			0.13 (0.005–3.42)	0.219
Undecided	0.47 (0.05–4.20)			0.24 (0.005–12.61)	0.479
Little	0.11 (0.01–1.07)			0.12 (0.002–6.33)	0.291
Least	0.02 (0.001–0.33)			0.001 (0.0005–1.67)	0.069

Abbreviations: OR, odds ratio; 95% CI, 95% confidence interval.

Interest in specific research topics was especially high for multimorbidity (33% “extremely high”) and hypertension (32%). Research designs and research methods were not that important and did not differ between subtypes. Most GPs rated research as “extremely important” (57%) for the future of primary care (S2 Table).

Seventeen GPs mentioned their own study ideas, with topics varying from pain therapy, vitamin D deficiency, to topics relating to compliance and insurance. Thirty-nine GPs mentioned already having participated in research projects. In terms of changes needed in their practice to participate in research, the most frequent replies were that GPs needed an increased workforce and a reduction in their administrative workload. A decrease in the number of patients per day was also mentioned as a prerequisite.

## Discussion

### Principal findings

Almost half of the GPs in the canton of Lucerne indicated willingness to participate in future research projects. There was an association between the willingness to participate and GPs’ young age, male gender, urban location, and working in a group practice; these trends were independent of each other.

### Strengths and limitations

The phone calls were an important step to include all GPs in the analysis, as the written survey alone would have resulted in a biased proportion of GPs who are willing to participate in research. A study in one canton in Switzerland makes generalizability difficult. However, GPs in most other regions of Switzerland were within the reach of an established Institute of Primary Care and therefore already confronted with research proposals, which would have been difficult to combine with our mostly unaffected population. Residual confounding might be present due to the non-randomized manner of the investigation; the absence of potential, unknown confounders; the way predictor variables were modeled in the regression; or most importantly the imperfect method via regression model to account for confounding. Furthermore, the stated willingness to participate in the survey does not necessarily correlate with actual behavior. The current study is about the general willingness and does not exclude the possibility that GPs participate if specific research projects are presented in more detail.

### Comparison with former publications

Previous studies have already shown that younger GPs and GPs working in larger practices were more likely to participate in research [12,16]. Comparing our result that male GPs are more willing to participate in research with previous studies is inconclusive, with confirming results in one study [12] and no influence of gender in other studies [16–18].

A study conducted in the UK, which compared research active and non-active practices showed an overrepresentation of research active practices in urban and deprived areas [16]. On the contrary, an Australian study showed that GPs working in outer suburban or rural practices had higher interests in research than their inner suburban and provincial city counterparts [17]. A French study found no difference due to practice location [18]. The reasons for these differences are not clear and need further investigation.

Previous evidence on the role of financial compensation for the willingness to participate in research is mixed. Some studies demonstrated that a monetary incentive has the effect that GPs are more willing to participate in research. Especially surveys were rather being answered by GPs if incentivized and the motivation to sacrifice their leisure time for research was higher with a financial compensation [19,20]. Studies showed that smaller practices and newly established practices may have difficulties to cover the expense of research studies [16,21,22]. In a German study, the authors found that financial compensation is rather unimportant [23]. In our study, few GPs indicated that money plays an important role. However, there was a trend that those few GPs were more willing to participate in research. We hypothesize that GPs in Southern Germany and Switzerland face a less tens financial situation compared to other countries. On the other hand, our result could also be related to the non-anonymized survey which led to GPs who did not answer the survey honestly; we therefore must assume that financial compensation plays a more important role than our results indicate.

The considerable differences in the acceptance of the different research topics is known from former publications. In general, research projects around personal motives of the GPs and highly relevant topics for daily practice are better recognized [21,24]. In our study, the topics of hypertension treatment and caring for multimorbid patients at home were relevant for the GPs, similar to former questionnaire studies where cardiovascular topics and chronic Illness were top priorities [18,19]. Other topics such as preventive medicine do not show a clear pattern, their acceptance as research topics might underlie temporal or regional trends [19].

### Implications of this study

The follow-up by telephone was strongly associated with a low willingness to participate in research. We assume that the phone call was less suitable to pique the interest in research than the postal questionnaire. The call might as well corroborate a negative response implied by GPs who did not respond to the postal questionnaire.

It will be important to choose topics that are of high relevance to the GPs and that time expenses are limited. Disinterest in participating in research was mainly justified by constraints of general practice, most frequently the lack of time, as previous studies have also shown [25–27]. Some GPs do not consider themselves part of a greater health care team and do not consider participation in research part of their clinical role, as has been observed in a study conducted in Germany [28]. Countries with a legal framework for interprofessional teams in primary care, such as the UK and Netherlands, might provide more options assigning staff to collaborate in research projects [13,14]. In Finland and Germany, large-scaled policy interventions such as quality improvement schemes accelerated the introduction of research-friendly electronic health records [8,9].

It is suggested that the involvement of government and national funding bodies, along with support from universities and primary care departments, are needed to build research networks and to develop international alliances to facilitate the international exchange of information [29]. This would help to simplify and align research among different countries.

### Outlook

The workload of the GPs could be relieved by facilitating data collection, such as with the help of the FIRE (Family Medicine ICPC-Research using Electronic Medical Records) project, which is a promising ongoing effort to establish research networks accessible for all GPs, including those in rural areas [30–33].

Furthermore, it would be important to accomplish a stronger interaction with GPs and point out the importance of their participation in primary care research, starting at university level involving students.

## Conclusion

Scientists should develop methods to motivate and support GPs in single and rural practices to participate so research is representative of all primary care physicians.

## Supporting information

S1 Dataset Anonymized dataset. (XLSX)

S1 Table Characteristics of participants and non-participants in survey. (DOCX)

S2 Table Respondents’ answers to questionnaire. (DOCX)

S1 Text Questionnaire in German (Original). (DOCX)

S2 Text Questionnaire in English (Translated). (DOCX)
