# Supplementary material for: General practitioners’ willingness to participate in research: A survey in central Switzerland
Source: PLoS One. 2019 Mar 1;14(3):e0213358. doi: 10.1371/journal.pone.0213358 (PMC6396922; doi:10.1371/journal.pone.0213358)
Supplement: S1 Table — (DOCX) [file pone.0213358.s002.docx]

**Supplementary Table S4. Characteristics of participants and non-participants in survey.**

| **Variable** | **Respondents (n=139)** | **Non-respondents (n=129)** |
| --- | --- | --- |
|  | **n (%)** | **n (%)** |
| Age (years)   - 33-40 - 41-50 - 51-60 - 61+ | 20 (15)  30 (22)  61 (45)  25 (18) | 11 (9)  29 (23)  57 (45)  30 (23) |
| Gender   - male - female | 40 (29)  99 (71) | 40 (31)  89 (69) |
| Type of practice   - single - group | 87 (63)  52 (37) | 70 (54)  59 (46) |
| Location of practice   - urban - rural | 84 (60)  55 (40) | 72 (56)  57 (44) |
