# Supplementary material for: General practitioners’ willingness to participate in research: A survey in central Switzerland
Source: PLoS One. 2019 Mar 1;14(3):e0213358. doi: 10.1371/journal.pone.0213358 (PMC6396922; doi:10.1371/journal.pone.0213358)
Supplement: S2 Table — (DOCX) [file pone.0213358.s003.docx]

**Supplementary Table S5. Respondents’ answers to questionnaire.**

| **Factors that influence participation in research topics:** | **Extremely important (1)** | **Very important (2)** | **Moderately important (3)** | **Slightly important (4)** | **Not at all important (5)** |
| --- | --- | --- | --- | --- | --- |
| Time constraints | 93 (67%) | 33 (24%) | 10 (7%) | 2 (2%) | 0 |
| Research topic | 79 (58%) | 47 (34%) | 5 (4%) | 4 (3%) | 1 (1%) |
| Integration in research network | 42 (31%) | 52 (38%) | 29 (21%) | 9 (7%) | 4 (3%) |
| Regular further training on research questions | 16 (12%) | 51 (37%) | 48 (35%) | 16 (12%) | 5 (4%) |
| Financial compensation | 6 (6%) | 24 (25%) | 41 (44%) | 14 (15%) | 9 (10%) |
| **Interest in the following research topics:** | **Extreme (1)** | **High (2)** | **Moderate (3)** | **Slight (4)** | **None at all (5)** |
| Prevention of pressure ulcer in immobilized patients | 8 (6%) | 24 (18%) | 47 (35%) | 32 (23%) | 25 (18%) |
| Taking care of patients with migration background | 20 (15%) | 42 (30%) | 39 (28%) | 20 (15%) | 17 (12%) |
| Anemia work-up | 33 (24%) | 54 (39%) | 34 (25%) | 10 (7%) | 7 (5%) |
| Treatment of hypertension | 44 (32%) | 46 (33%) | 29 (21%) | 12 (9%) | 7 (5%) |
| Care of multimorbid patients with at home | 45 (33%) | 41 (30%) | 28 (20%) | 14 (10%) | 9 (7%) |
| Supportive use of placebo for treatment of chronic pain | 20 (15%) | 37 (27%) | 37 (27%) | 24 (17%) | 19 (14%) |
| Influence of doctor-patient relationship on the course of chronic disease | 39 (28%) | 47 (34%) | 34 (25%) | 11 (8%) | 7 (5%) |
| **Interest in participating in a research project in the following categories:** | **Extreme (1)** | **High (2)** | **Moderate (3)** | **Slight (4)** | **None (5)** |
| Diagnostic studies | 26 (19%) | 60 (44%) | 28 (21%) | 12 (9%) | 10 (7%) |
| Therapeutic studies | 22 (16%) | 52 (38%) | 33 (24%) | 20 (15%) | 10 (7%) |
| Development of guidelines for patient pathways and recommendations | 28 (20%) | 41 (30%) | 37 (27%) | 22 (16%) | 10 (7%) |
| **Interest in the following research methodologies:** | **Extreme (1)** | **High (2)** | **Moderate (3)** | **Slight (4)** | **None at all (5)** |
| Qualitative studies | 19 (14%) | 49 (36%) | 41 (30%) | 20 (15%) | 7 (5%) |
| Intervention studies | 17 (12.5%) | 53 (39%) | 43 (32%) | 17 (12.5%) | 6 (4%) |
| Observational study | 17 (12.5%) | 63 (46%) | 35 (25.5%) | 15 (11%) | 7 (5%) |
| **Importance of primary care research for family medicine’s future** | 55 (57%) | 30 (31%) | 10 (10%) | 1 (1%) | 0 |
