# Supplementary material for: General practitioners’ willingness to participate in research: A survey in central Switzerland
Source: PLoS One. 2019 Mar 1;14(3):e0213358. doi: 10.1371/journal.pone.0213358 (PMC6396922; doi:10.1371/journal.pone.0213358)
Supplement: S1 Text — (DOCX) [file pone.0213358.s004.docx]

Kontakt: stefan.essig@iham-cc.ch

# Forschung in der Hausarztpraxis

Teilnehmer: Dr. med. Vorname Nachname, Ort

1. Wie wichtig finden Sie Forschung für die Zukunft der Hausarztmedizin? (bitte ankreuzen)

| Sehr  ++ | Ziemlich  + | Neutral  o | Wenig  - | Gar nicht  -- |
| --- | --- | --- | --- | --- |

1. Das Institut für Hausarztmedizin und Community Care Luzern bereitet einige Forschungsthemen vor. Wie sehr würden Sie sich grundsätzlich dafür interessieren, an einer Forschungsarbeit zu folgenden Themen teilzunehmen?

Prävention von **Dekubitus** bei **immobilisierten** Patienten

| Sehr  ++ | Ziemlich  + |  | Neutral  o | Wenig  - | Gar nicht  -- |
| --- | --- | --- | --- | --- | --- |
| Betreuung von **Migranten** in der Praxis | | |  |  |  |
| Sehr  ++ | Ziemlich  + |  | Neutral  o | Wenig  - | Gar nicht  -- |
| **Anämieabklärung** in der Praxis | | |  |  |  |
| Sehr  ++ | Ziemlich  + |  | Neutral  o | Wenig  - | Gar nicht  -- |
| **Hypertoniebehandlung** in der Praxis | | |  |  |  |
| Sehr  ++ | Ziemlich  + |  | Neutral  o | Wenig  - | Gar nicht  -- |
| Betreuung **multimorbider** Patienten zu Hause | | | |  |  |
| Sehr  ++ | Ziemlich  + | Neutral  o | | Wenig  - | Gar nicht  -- |
| Unterstützender Einsatz von **Placebo bei chronischen Schmerzen** | | | | |  |
| Sehr  ++ | Ziemlich  + | Neutral  o | | Wenig  - | Gar nicht  -- |

Einfluss der **Arzt-Patienten-Beziehung** auf den Verlauf chronischer Erkrankungen

| Sehr  ++ | Ziemlich  + | Neutral  o | Wenig  - | Gar nicht  -- |
| --- | --- | --- | --- | --- |

1. Wie sehr würden Sie sich grundsätzlich dafür interessieren, an einer Forschungsarbeit zu folgenden Kategorien teilzunehmen?

**Diagnostische** Studien, z.B. Korrelation eines Laborwerts mit spezifischen Symptomen

| Sehr  ++ | Ziemlich  + | Neutral  o | Wenig  - | Gar nicht  -- |
| --- | --- | --- | --- | --- |

**Therapeutische** Studien, z.B. Vergleich der Wirksamkeit zweier Medikamente

| Sehr  ++ | Ziemlich  + | Neutral  o | Wenig  - | Gar nicht  -- |
| --- | --- | --- | --- | --- |

Entwicklung von **Guidelines** zu bestimmten Abklärungen oder Therapieempfehlungen

| Sehr  ++ | Ziemlich  + | Neutral  o | Wenig  - | Gar nicht  -- |
| --- | --- | --- | --- | --- |

1. Welche Art von **Forschungsmethodik** würde Ihnen bei einer Teilnahme insbesondere zusagen?

**Qualitative Studie**, z.B. Diskussionsrunde mit anderen Hausärzten oder Interview

| Sehr  ++ | Ziemlich + | Neutral  o | Wenig  - |  | Gar nicht  -- |
| --- | --- | --- | --- | --- | --- |
| **Interventionsstudie**, z.B. spezifische Beratung bei Diabetikern vs. Usual Care | | | | |  |
| Sehr  ++ | Ziemlich + | Neutral  o | Wenig  - |  | Gar nicht  -- |

**Beobachtungsstudie**, z.B. Häufigkeit verschiedener Anämieursachen in einer Patientengruppe

| Sehr  ++ | Ziemlich  + | Neutral  o | Wenig  - | Gar nicht  -- |
| --- | --- | --- | --- | --- |

1. Haben Sie eigene Studienideen? Bitte fassen Sie diese in Form eines Titels zusammen:

_____________________________________________________________________________________________

_____________________________________________________________________________________________

1. Wie sehr spielen neben dem grundsätzlichen Interesse **folgende Faktoren** eine Rolle, ob Sie an der Forschungsarbeit teilnehmen?

Finanzielle Entschädigung

| Sehr  ++ | Ziemlich  + | Neutral o | Wenig  - | Gar nicht  -- |
| --- | --- | --- | --- | --- |
| Zeitlicher Aufwand | |  |  |  |
| Sehr  ++ | Ziemlich  + | Neutral  o | Wenig  - | Gar nicht  -- |
| Thema | |  |  |  |
| Sehr  ++ | Ziemlich  + | Neutral  o | Wenig  - | Gar nicht  -- |

Einbindung in aktives Forschungsnetz mit gewährleistetem Support durch das Institut

| Sehr  ++ | Ziemlich  + | Neutral  o | Wenig  - | Gar nicht  -- |
| --- | --- | --- | --- | --- |
| Regelmässige Fortbildungen zu Forschungsfragen | | |  |  |
| Sehr  ++ | Ziemlich  + | Neutral  o | Wenig  - | Gar nicht  -- |

Andere: ____________________________________________________________________

1. Dürften wir Sie **grundsätzlich** für die Teilnahme an künftigen Forschungsprojekten kontaktieren?

| Ja | Nein |
| --- | --- |

1. Dürfen wir Sie konkret für die Teilnahme an einer **Interventionsstudie zum Thema Steroideinnahme und Exazerbationen von COPD** kontaktieren?

| Ja | Nein |
| --- | --- |

1. Haben Sie bisher als Hausarzt an Forschungsprojekten teilgenommen? Wenn ja, an welchen Projekten?

_____________________________________________________________________________________________

_____________________________________________________________________________________________
