# Supplementary material for: General practitioners’ willingness to participate in research: A survey in central Switzerland
Source: PLoS One. 2019 Mar 1;14(3):e0213358. doi: 10.1371/journal.pone.0213358 (PMC6396922; doi:10.1371/journal.pone.0213358)
Supplement: S2 Text — (DOCX) [file pone.0213358.s005.docx]

Contact: stefan.essig@iham-cc.ch

# Research in Primary Care

Participant: Dr. med. *First name, Last name, place*

1. How important do you consider research for the future of primary care?

| Very  ++ | Quite + | Neutral  o | Little  - | Not at all  -- |
| --- | --- | --- | --- | --- |

1. The Institute of Primary and Community Care Lucerne is currently preparing its research agenda. How much would you be interested in participating in a research project on the following topic?

Prevention of pressure ulcer in immobilized patients

| Very  ++ | Quite + | Neutral  o | | Little  - | Not at all  -- |
| --- | --- | --- | --- | --- | --- |
| Taking care of patients with migration background in the practice | | | | |  |
| Very  ++ | Quite + |  | Neutral  o | Little  - | Not at all  -- |
| Anemia work-up in the practice | | |  |  |  |
| Very  ++ | Quite + |  | Neutral  o | Little  - | Not at all  -- |
| Therapy of hypertenstion in the practice | | |  |  |  |
| Very  ++ | Quite + |  | Neutral  o | Little  - | Not at all  -- |
| Caring for multimorbid patients at home | | |  |  |  |
| Very  ++ | Quite + |  | Neutral  o | Little  - | Not at all  -- |
| Supportive use of placebo for treatment of chronic pain | | | |  |  |
| Very  ++ | Quite + | Neutral  o | | Little  - | Not at all  -- |
| Influence of doctor-patient-relationship on the course of chronic disease | | | | |  |
| Very  ++ | Quite + | Neutral  o | | Little  - | Not at all  -- |

1. How much would you generally be interested in participating in a research project of the following categories?

Diagnostic studies, for example correlation of laboratory values with specific symptoms

| Very  ++ | Quite + | Neutral  o | Little  - | Not at all  -- |
| --- | --- | --- | --- | --- |
| Therapeutic studies, for example, comparing the efficacy of two drugs | | | |  |
| Very  ++ | Quite + | Neutral  o | Little  - | Not at all  -- |
| Developing guidelines for patient pathways and recommendations | | | |  |
| Very  ++ | Quite + | Neutral  o | Little  - | Not at all  -- |

1. Which type of research methods would you particularly be interested in?

Qualitative study, for example, panel discussion with other general practitioners or interviews

| Very  ++ | Quite + | Neutral  o | Little  - | Not at all  -- |
| --- | --- | --- | --- | --- |

Intervention study, for example, specific chronic care for diabetics versus usual care

| Very  ++ | Quite + | Neutral  o | Little  - | Not at all  -- |
| --- | --- | --- | --- | --- |
| Observational study, for example, incidence of different causes of anemia | | | |  |
| Very  ++ | Quite + | Neutral  o | Little  - | Not at all  -- |

1. Do you have your own study ideas? Please summarize them as a title:

_____________________________________________________________________________________________

_____________________________________________________________________________________________

1. How relevant are the following factors to participate in research besides your general interest?

Financial compensation

| Very  ++ | Quite + | Neutral  o | Little  - | Not at all  -- |
| --- | --- | --- | --- | --- |
| Time involved |  |  |  |  |
| Very  ++ | Quite + | Neutral  o | Little  - | Not at all  -- |
| Topic |  |  |  |  |
| Very  ++ | Quite + | Neutral  o | Little  - | Not at all  -- |

Integration into an active research network with guaranteed support by the institute

| Very  ++ | Quite + | Neutral  o | Little  - | Not at all  -- |
| --- | --- | --- | --- | --- |
| Regular further trainings on research questions | | |  |  |
| Very  ++ | Quite + | Neutral  o | Little  - | Not at all  -- |

Andere: ____________________________________________________________________

1. In general, can we contact you for participation in future research projects?

| Yes | No |
| --- | --- |

1. In specific, can we contact you for participation in an intervention study on intake of steroids and exacerbations of COPD?

| Yes | No |
| --- | --- |

1. Have you already participated in research project as general practitioner? If yes, which projects?

_____________________________________________________________________________________________

_____________________________________________________________________________________________
